# Effect of exercise and/or reduced calorie dietary interventions on breast cancer-related endogenous sex hormones in healthy postmenopausal women

**DOI:** 10.1186/s13058-018-1009-8

**Published:** 2018-08-02

**Authors:** Martijn de Roon, Anne M. May, Anne McTiernan, Rob J. P. M. Scholten, Petra H. M. Peeters, Christine M. Friedenreich, Evelyn M. Monninkhof

**Affiliations:** 1 Department of Epidemiology, Julius Center for Health Sciences and Primary Care, University Medical Center Utrecht, PO Box 85500, 3508 GA Utrecht, the Netherlands; 2 Epidemiology Program, Division of Public Health Sciences, Fred Hutchinson Cancer Research Centre, Seattle, Washington USA; 3 Department of Epidemiology, School of Public Health, and Department of Medicine, School of Medicine, University of Washington, Seattle, Washington USA; 4 Cochrane Netherlands, University Medical Center Utrecht, Utrecht, the Netherlands; 5 MRC-PHE Centre for Environment and Health, Department of Epidemiology and Biostatistics, School of Public Health, Imperial College, London, UK; 6 Department of Cancer Epidemiology and Prevention Research, CancerControl Alberta, Alberta Health Services, Alberta, Canada; 7 Department of Oncology, Cumming School of Medicine, University of Calgary, Calgary, Canada; 8 Department of Community Health Sciences, Cumming School of Medicine, University of Calgary, Calgary, Canada

**Keywords:** Breast cancer, Postmenopausal women, Exercise, Caloric restriction, Prevention, Sex hormones, Weight loss

## Abstract

**Background:**

Physical inactivity and being overweight are modifiable lifestyle risk factors that consistently have been associated with a higher risk of postmenopausal breast cancer in observational studies. One biologic hypothesis underlying this relationship may be via endogenous sex hormone levels. It is unclear if changes in dietary intake, physical activity, or both, are most effective in changing these hormone levels.

**Objective:**

This systematic review and meta-analysis examines the effect of reduced caloric dietary intake and/or increased exercise levels on breast cancer-related endogenous sex hormones.

**Methods:**

We conducted a systematic literature search in MEDLINE, Embase, and Cochrane’s Central Register of Controlled Trials (CENTRAL) up to March 2017. Main outcome measures were breast cancer-related endogenous sex hormones.

Randomized controlled trials (RCTs) reporting effects of reduced caloric intake and/or exercise interventions on endogenous sex hormones in healthy, physically inactive postmenopausal women were included. Studies including women using hormone therapy were excluded. The methodological quality of each study was assessed by the Cochrane’s risk of bias tool.

**Results:**

From the 2599 articles retrieved, seven articles from six RCTs were included in this meta-analysis. These trials investigated 1588 healthy postmenopausal women with a mean age ranging from 58 to 61 years. A combined intervention of reduced caloric intake and exercise, with durations ranging from 16 to 52 weeks, compared with a control group (without an intervention to achieve weight loss) resulted in the largest beneficial effects on estrone treatment effect ratio (TER) = 0.90 (95% confidence interval (CI) = 0.83–0.97), total estradiol TER = 0.82 (0.75–0.90), free estradiol TER = 0.73 (0.66–0.81), free testosterone TER = 0.86 (0.79–0.93), and sex hormone biding globulin (SHBG) TER = 1.23 (1.15–1.31). A reduced caloric intake without an exercise intervention resulted in significant effects compared with control on total estradiol TER = 0.86 (0.77–0.95), free estradiol TER = 0.77 (0.69–0.84), free testosterone TER = 0.91 (0.84–0.98), and SHBG TER = 1.20 (1.06–1.36). Exercise without dietary change, versus control, resulted in borderline significant effects on androstenedione TER = 0.97 (0.94–1.00), total estradiol TER = 0. 97 (0.94–1.00), and free testosterone TER = 0. 0.97 (0.95–1.00).

**Conclusions and relevance:**

This meta-analysis of six RCTs demonstrated that there are beneficial effects of exercise, reduced caloric dietary intake or, preferably, a combination of exercise and diet on breast cancer-related endogenous sex hormones in physically inactive postmenopausal women.

**Electronic supplementary material:**

The online version of this article (10.1186/s13058-018-1009-8) contains supplementary material, which is available to authorized users.

## Background

Breast cancer is the most common invasive cancer among women worldwide with 1.67 million new cases diagnosed in 2012 [1]. Although numerous breast cancer risk factors are known, most are not easily amenable to intervention. Low levels of physical activity and being overweight are modifiable lifestyle risk factors for breast cancer that have been consistently associated with a higher risk of postmenopausal breast cancer in observational epidemiologic studies [2–5]. One of the pathways, with one of the largest bodies of evidence, is via endogenous sex hormones [6].

High levels of sex serum hormones, including estrogens and androgens, and low levels of sex hormone binding globulin (SHBG) are associated with higher postmenopausal breast cancer risk [4, 7]. SHBG binds to estradiol and testosterone and thereby reduces their harmful free fractions [8, 9]. In postmenopausal women, the main source of estrogens and androgens is via conversion of precursors in peripheral fat tissue [10, 11]. Postmenopausal women who are overweight and/or physically inactive have been shown to have higher levels of circulating endogenous sex hormones [12, 13].

Physical activity might affect sex hormonal levels by reducing the amounts of adipose tissue [14–16]. Normal-weight women show lower levels of estrogens and higher levels of SHBG causing decreased levels of free estradiol compared with overweight/obese women [14–16]. Two large multi-armed randomized controlled trials (RCTs) (*n* = 439 and *n* = 243, respectively) have also shown that weight loss and fat loss can be achieved by reduced caloric intake, also affecting sex hormonal levels [17, 18]. However, it is unclear what the most effective method is to reduce postmenopausal endogenous sex hormones.

The aim of this systematic review was to summarize the evidence and to compare the effectiveness of reduced caloric intake and/or exercise on endogenous sex steroid hormones in postmenopausal women.

## Methods

In February 2018 we searched MEDLINE, Embase, and the Cochrane Central Register of Controlled Trials (CENTRAL) for eligible studies. The following MeSH terms, keywords, and synonyms of those terms were used: physical activity, exercise, weight loss, diet, postmenopausal, sex hormones. A more detailed description of the search strategies is presented in Additional file 1. We additionally checked the references of the included studies.

This meta-analysis was registered in Prospero, the international register of systematic reviews, with registration number CRD42015026094.

### Selection of studies

Study selection was performed by two authors (MdR, EMM) independently. Studies were first screened on title. After screening on title, a second screening on the remaining potentially eligible abstracts was performed. Of potentially eligible studies, definite selection was based on a full-text copy of the study. Disagreements between the two authors were resolved by discussion. If no consensus could be achieved, a third author (AMM) was consulted.

We included RCTs comparing a reduced calorie dietary intervention, an exercise intervention, or both, with each other or with a control group in healthy postmenopausal women with endogenous sex hormones as outcome measurements. In this meta-analysis, trial arms were considered controls if they did not receive any form of intervention or received only a stretching/relaxation program. Furthermore, studies were excluded when the study population consisted of women using hormone therapy, contained less than 20 women, or the intervention period was less than 12 weeks (since physiologically it is unlikely to expect a meaningful reduction in adipose tissue, which is one mechanism by which physical activity affects sex hormone levels, in such a short time frame [19]).

### Data extraction

One author (MdR) extracted data using a predefined data extraction form. Data extracted included: 1) author(s), year, study nationality; 2) details of the study design, size, study duration; 3) characteristics of the study population (age, bodyweight, body mass index (BMI), etc.); 4) details of the interventions; and 5) study results. Extractions of study results were checked by a second author (EMM). For data extraction and quality assessment, both the paper and, if available, the study protocol were used. If data were missing or further information was required, we contacted the study authors to request further information.

### Quality assessment

A risk of bias assessment was performed with Cochrane’s risk of bias tool by two authors (MdR and EMM) independently [20]. This tool addresses the following domains: 1) randomization; 2) concealment of allocation; 3) blinding of participants and personnel; 4) blinding of outcome assessment; 5) incomplete outcome data; 6) selective outcome reporting; and 7) other biases. Each item was scored as low, unclear, or high risk of bias. For other biases, three topics were scored: were blood samples of the same women analyzed in the same batch, were the participants instructed to avoid exercise 24 h before blood sampling, and was adherence to the exercise and/or reduced calorie diet program monitored. If one or more of these three topics was not met studies were scored as high risk of bias for this item. When information regarding these potential sources of bias was missing in the publication or the study protocol, the study authors were contacted.

### Data synthesis and analysis

We analyzed the data for six comparisons: 1) exercise intervention versus control; 2) combined exercise and reduced calorie diet versus no intervention; 3) reduced calorie diet versus no intervention; 4) combined exercise and reduced calorie diet versus reduced calorie diet alone; 5) combined exercise and reduced calorie diet versus exercise alone; and 6) exercise intervention versus reduced calorie diet. When at least two studies were available for a comparison and no substantial heterogeneity was present, meta-analysis was performed according to the generic inverse variance method by the use of Cochrane’s Review Manager (RevMan®) version 5.3.5 [21]. Studies reported either geometric means of sex hormone levels at the end of the study or a treatment effect ratio (TER) (i.e., the ratio of the geometric means of the study arms). For meta-analysis, we used the log-transformed value of these measures. For log(TER), we derived the standard error (SE) from the 95% confidence interval (CI) of TER. When geometric means were reported per study arm, we calculated the difference of the log-transformed geometric means (which is equal to the log(TER)) and derived the SE of the log(TER) from the 95% CIs of the geometric means of the respective study arms. For calculation of these values, we used the built-in calculator of RevMan. If the required values were not reported, we contacted the study authors. All tables and figures in this meta-analysis are original for this article.

For each meta-analysis, a random effects model was used. Heterogeneity between studies was assessed by visual inspection of the forest plots (i.e., whether confidence intervals overlap), the Chi-square test for homogeneity, and the *I*^2^ statistic. Values of 25%, 50%, and 75% indicate low, moderate, and high heterogeneity, respectively.

## Results

**Fig. 1:**
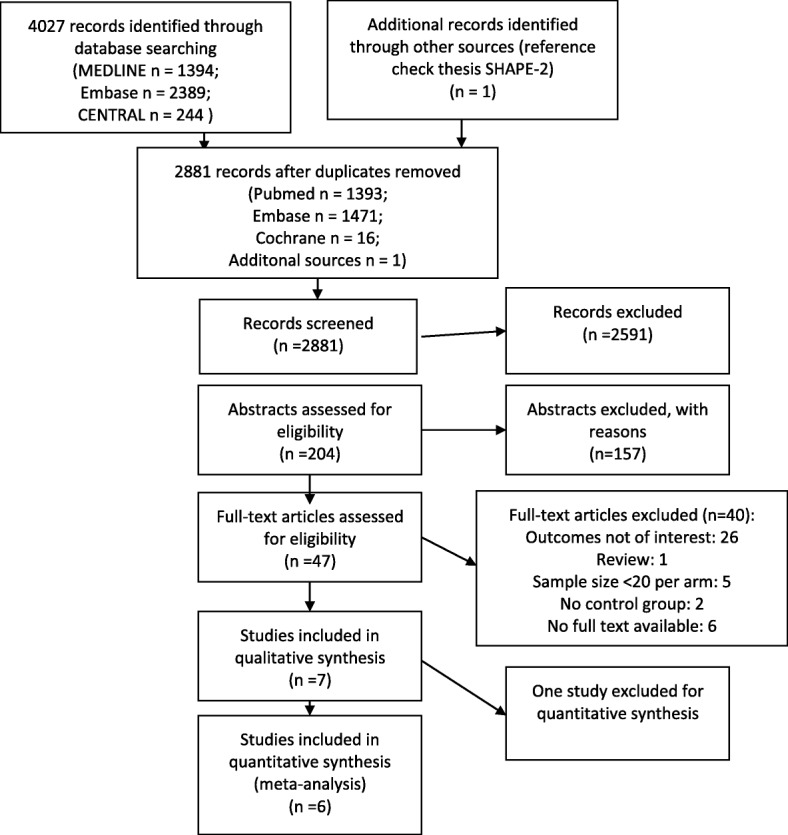
Flow chart of the selection and inclusion of eligible studies

The search resulted in 4027 articles (Fig. 1). After removing duplicates, 2881 references remained. After screening titles and abstracts, 47 references remained for full text screening. Of these, 40 references were excluded. Reasons for exclusion were: did not address our outcomes of interest (*n* = 26), sample size per study arm was < 20 (*n* = 5), no full text available (*n* = 6), no control group (control group was offered an intervention other than stretching/relaxation; *n* = 2), study was not an original (randomized) trial (*n* = 1). Finally, we included seven articles from six randomized controlled trials [3, 17, 18, 22–25].

The main characteristics of the six included studies are shown in Tables 1 and 2. These studies were published between 2004 and 2015, and investigated a total of 1588 postmenopausal women with a mean age ranging from 57.8 to 61.2 years. Five of the six studies included a control group that did not receive any intervention. The control group of the Physical Activity for Total Health (PATH) trial received a stretching program (that was not assumed to affect weight loss or measures of fitness) [22, 23]. Two studies compared two or three interventions with control.

**Table 1:** Characteristics of the six included studies

Study	Study arms	Mean age (SD) (years)	Sample size, *n*; drop out, *n* (%)	Sex hormone outcomes	Methods for sex hormone evaluation	Intervention period
SHAPE-2 [18], 2015, The Netherlands	Ex + D	59.5 (4.9)	98; 9 (9%)	Total estradiol, estrone, free estradiol, total testosterone, free testosterone, SHBG, androstenedione	Determined by liquid chromatography-mass spectrometry (LC-MC). SHBG by double-antibody radioimmunoassay (RIA) kits^b^	16 weeks^a^
	D	60.5 (4.6)	97; 6 (6%)			
	C	60.0 (4.9)	48; 3 (6%)			
NEW trial [17], 2012, United States	Ex + D	58.0 (4.4)	117; 9 (8%)	Total estradiol, estrone, free estradiol, total testosterone, free testosterone, SHBG, androstenedione	Quantified by RIA after organic solvent extraction and Celite column partition chromatography. SHBG via chemiluminescent immunometric assay using Immulite Analyzer^b^	6 months, 12 months
	Ex	58.1 (5.0)	117; 11 (9%)			
	D	58.1 (5.9)	118; 13 (11%)			
	C	57.4 (4.4)	87; 7 (8%)			
ALPHA trial [24], 2010, Canada	Ex	61.2 (5.4)	160; 6 (4%)	Total estradiol, estrone, free estradiol, total testosterone, free testosterone, SHBG, androstenedione	Quantified by RIA after organic solvent extraction and Celite column partition chromatography. SHBG via immunometric assay using Immulite Analyzer^b^	6 months, 12 months
	C	60.6 (5.7)	160; 6 (4%)			
SHAPE-1, 2009 [3], The Netherlands	Ex	58.9 (4.6)	96; 1 (1%)	Total estradiol, estrone, free estradiol, total testosterone, free testosterone, SHBG, androstenedione	Double-antibody RIA kits were used for determining sex hormones, also for SHBG^b^	4 months, 12 months
	C	58.4 (4.2)	93; 5 (5%)			
Orsatti et al. [25], 2008, Brazil	Ex	57.8 (8.0)	27; 6 (22%)	Total testosterone, total estradiol	Measured by the Immulite System, automated immunoassay.	16 weeks
	C	59.3 (6.2)	23; 1 (4%)			
PATH trial [22, 23], 2004, United States	Ex	60.7 (6.7)	87; 3 (3%)	Total estradiol, estrone, free estradiol, total testosterone, free testosterone, SHBG, androstenedione	Quantified by RIA after organic solvent extraction and Celite column partition chromatography. SHBG via immunometric assay using Immulite Analyzer^b^	12 months
	C	60.6 (6.8)	86; 0			

*ALPHA* Alberta Physical Activity and Breast Cancer, *C* control, *D* reduced calorie diet, *Ex* exercise, *NEW* Nutrition and Exercise for Woman, *PATH* Physical Activity for Total Health, *SHAPE* Sex Hormone and Physical Exercise, *SHBG* sex hormone binding globulin

^a^Although this study took 16 weeks, results were pooled with the other studies

^b^Free estradiol/free testosterone were calculated using the measured values for estradiol, testosterone, and SHBG, and assumed constant for albumin

**Table 2:** Intervention characteristics of included studies

Study	Intervention	Start intervention	Duration of program	Frequency and duration of sessions	Intensity	Adherence	Control group
SHAPE-2 [18], 2015	Ex + D D	4–6 week run-in period. Standardized diet to maintain stable weight and to achieve a comparable diet composition among all participants	4–6 run period + 16 week intervention	4 h/week, two 1-h group sessions of combined strength and endurance, two 1-h sessions of Nordic walking Two individual consultations of 30 min with dietician. Five 1-h group sessions spread over the study period	20–25 min of endurance training (60–90% of HRR), 25 min strength training, 5–10 min WU/CD, caloric restriction was 1750 kcal/week. Moderate to vigorous Nordic walking (60–65% of HRR) Diet group was prescribed a caloric restriction of 3500 kcal/week (or 500 kcal/day)	Group sessions were supervised. Participants kept an exercise log that the physiotherapist regularly checked Telephone calls every other week	The control group was asked to maintain a stable weight by continuing the standardized diet and their habitual physical activity patterns. After study completion the control group was offered a weight loss intervention
NEW trial [17], 2012	Ex D Ex + D		A 10% reduction in body weight at 6 months with maintenance thereafter to 12 months	5 days/225 min per week for 12 months. Weekly group meetings with a dietician for the first 6 months, thereafter dieticians contacted participants twice a month, including one face to face contact and one additional phone call or e-mail. Women in the diet + exercise group received both interventions	Exercise started with a 15 min session at 60–70% of MHR and progressed to 70–85% of MHR for 45 min by the 7th week where it was maintained for the rest of the study. The dietary intervention comprised a modification of the dietary component of the dietary prevention program and Look Ahead lifestyle intervention programs, with the following goals: total daily energy intake of 1200–2000 kcal/day based on baseline weight, less than 30% daily intake from fat, and a 10% reduction in bodyweight, Women in the diet + exercise group received both interventions	Participants attended at least three supervised sessions per week. In home sessions they recorded mode and duration of the exercise. Women were asked to record all food eaten daily for at least 6 months. Journaling, weekly weighing, and sessions attendance were tracked to promote adherence	The control group was asked not to change their diet or exercise habits. After study completion the control group was offered a weight loss intervention
ALPHA trial [24], 2010	Ex	Started with three sessions per week of 15 to 20 min	During the first 3 months the frequency, duration and intensity increased and maintained for 9 more months	5 days per week of at least 45 min of aerobic exercise	During the first 3 months frequency, duration and intensity were increased from three sessions a week of 15 to 20 min in duration at an intensity of 50 to 60% of HRR to five sessions per week of at least 45 min at 70–80% of HRR	Three sessions per week were facility based. Adherence was monitored through weekly participant- and trainer- administered exercise logs	Controls were asked to maintain their inactive lifestyle. All participants were instructed not to change their usual diet
SHAPE-1 [3], 2009	Ex		12 months	Twice per week a 1-h exercise intervention, once per week home-based exercise	10 min WU, 25 min moderate to vigorous aerobic exercise at 60–85% of MHR, 25 min strength training, 5 min CD. Home-based exercise session contained 30 min of brisk walking or cycling with an intensity of moderate to vigorous intensity (60–80% of MHR)	Sport instructors register the attendance of the subjects. Study coordinator performed visits per exercise group to control adherence of the protocol	Controls were requested to retain their habitual exercise pattern
Orsatti et al. [25], 2008	Ex	Before training, subjects in the exercise group attended a 4-week adaptation period to become familiarized with the protocol	16 weeks	3 weekly session on nonconsecutive days, under supervision.	Initially lighter loads were used and subjects performed 1 set of 15 repetitions at 40–50% of 1-RM, progression was gradual till 3 sets of 8–12 repetitions at 60–80% of 1-RM were performed. Protocol consisted of dynamic exercises for both lower and upper limbs for a total of 50–60 min. Loads were periodically adjusted at the end of each month	Attendance was recorded by the trainers	Controls were advised to keep their habitual diets and asked not to change their exercise habits
PATH trial [22, 23], 2004	Ex		12 months	At least 45 min of moderate-intensity exercise, 5 days/week for 12 months	The training program started at 40% of observed MHR for 16 min/session and gradually increased to 60–75% of MHR for 45 min/session by week 8	Participants were required to attend the three offered supervised sessions/week during months 1–3 and to exercise on 2 days/week at home. For months 4–12, they were required to attend at least one of the three offered sessions/week at a study facility and to exercise 4 days per week at home or at the facility	Control participants attended 1 weekly 45-min stretching session for 12 months and were asked not to change other exercise habits. Both groups were asked to maintain their usual diet

*1-RM* one-repetition maximum, *ALPHA* Alberta Physical Activity and Breast Cancer, *CD* cooling down, *D* reduced calorie diet, *Ex* exercise, *HRR* heart rate reserve, *MHR* maximal heart rate, *NEW* Nutrition and Exercise for Woman, *PATH* Physical Activity for Total Health, *SHAPE* Sex Hormone and Physical Exercise, *WU* warming-up

A summary of the risk of bias of the included studies is presented in Additional file 2. We scored five studies as high quality [3, 17, 18, 23, 24] and one as low quality [25]. All studies scored high risk of bias on blinding of personnel since blinding of personnel was not applicable during the exercise interventions.

### Interventions

The six studies applied a range of intervention programs varying in duration from 16 weeks to 12 months (Tables 1 and 2). Five studies reported supervised sessions in their exercise program [3, 17, 18, 22–24]. The frequency of the exercise sessions varied from 2 to 5 days per week. The exercise sessions consisted of a warm up of 5–10 min and aerobic exercises guided by the maximum heart rate (MHR) or heart rate reserve (HRR) while intensity increased during the intervention program. The duration of aerobic exercises varied from 15 to 45 min per session. Most exercise interventions started with approximately the same intensity, 50–60% of MHR or HRR. Only the PATH trial intervention started at 40% MHR [22, 23]. Intensity at the end of the aerobic intervention period ranged between 70 and 90% of MHR or HRR in all studies. The Sex Hormone and Physical Exercise (SHAPE) 1 and 2 studies and the study by Orsatti et al. also included strength training in the exercise program [3, 18, 25].

Both SHAPE-2 and the Nutrition and Exercise for Woman (NEW) trial reduced calorie intake interventions and had specific weight loss goals. SHAPE-2 aimed for 5–6 kg of weight loss in both intervention groups (exercise group and exercise + diet group) while the NEW trial’s reduced calorie intake arms aimed for a 10% reduction in body weight at 6 months with maintenance thereafter to 12 months. In the SHAPE-2 trial, the diet group was prescribed a caloric restriction of 3500 kcal/week (or 500 kcal/day). In the NEW trial, the dietary intervention comprised a modification of the dietary component of the Diabetes Prevention Program [26, 27] and Look Ahead lifestyle intervention programs [27, 28], with the following goals: total daily energy intake of 1200–2000 kcal based on baseline weight, less than 30% daily intake from fat, and a 10% reduction in body weight.

### Body weight

All studies measured the effect of the intervention on weight or BMI. As shown in Table 3, the SHAPE-2 and NEW trials found the greatest amount of weight loss within the diet (SHAPE-2 −4.9%, NEW −9.1%) and exercise *+* diet group (SHAPE-2 −5.5%, NEW −9.8%) [17, 18]. The exercise groups (not intended to lose weight) in the SHAPE-1 study (−1.4%), the PATH trial (−1.6%), and the Alberta Physical Activity and Breast Cancer (ALPHA) trial (−2.3%) achieved modest decreases in weight and BMI [3, 17, 24]. The study by Orsatti et al. showed a small increase in body weight in the exercise group (+0.6%) [25].

**Table 3:** Effect of the interventions on body weight and on serum sex hormones levels

Name, duration	Baseline value^a^	Postintervention^a^	Within-group difference (%)	Between-group difference^b^	Between-group difference^b^
Weight (kg) or BMI^c^					
SHAPE-2 [18], 2015, 16 weeks					
Exercise + diet (Ex+WL)	80.4	74.9	−5.5	−5.58 (−6.32 to −4.84)	Ex+WL vs WL
Reduced calorie diet (WL)	80.3	75.4	−4.9	− 4.95 (−5.69 to −4.21)	−0.63 (−1.23 to −0.04)
Control	80.4	80.4	0.1	Referent	
NEW trial [17], 2012, 12 months					
Exercise + diet	82.5	72.7	−9.8	*P* < 0.001	Ex+WL vs WL, *P* = 0.1
Exercise	83.7	80.9	−2.8	*P* = 0.02	Ex+WL vs Ex, *P* < 0.001
Reduced calorie diet	84.0	74.9	−9.1	*P* < 0.001	Ex vs WL, *P* < 0.001
Control	84.2	83.7	−0.5	Referent	
ALPHA trial [24], 2010, 12 months					
Exercise			−2.3	−1.80 (−2.60 to −1.00)	
Control			−0.5	Referent	
SHAPE-1 [3], 2009, 12 months					
Exercise	73.6	72.2	−1.4	N/A	
Control	74.8	74.0	−0.8		
Orsatti et al. [25], 2008, BMI, 16 weeks					
Exercise	28.8^c^	29.6^c^	0.6^c^	*P* = 0.57	
Control	27.6^c^	27.1^c^	−0.5^c^	Referent	
PATH trial [22, 23], 2004, 12 months					
Exercise	81.6	80.3	−1.6	*P* = 0.1	
Control	81.7	81.8	0.1	Referent	
Total estradiol (pg/ml)					
SHAPE-2 [18], 2015					
Exercise + diet	3.69	3.22	−12.7	0.83 (0.73–0.95)	Ex+WL vs WL
Reduced calorie diet	4.20	3.62	−13.8	0.86 (0.75–0.98)	0.97 (0.87–1.08)
Control	3.89	4.01	3.11	Referent	
NEW trial [17], 2012					
Exercise + diet	11.5	9.2	−20.3	*P* < 0.001	Ex+WL vs WL, *P* = 0.07
Exercise	11.5	11.0	−4.9	*P* = 0.1	Ex+WL vs Ex, *P* < 0.001
Reduced calorie diet	11.6	9.7	−16.2	*P* < 0.001	Ex vs WL, *P* = 0.002
Control	10.9	11.4	4.9	Referent	
ALPHA trial [24], 2010					
Exercise	10.1	8.7		0.93 (0.88–0.98)	
Control	10.2	9.9		Referent	
SHAPE-1 [3], 2009					
Exercise	8.8	8.1	−7.3	0.99 (0.95–1.02)	
Control	9.8	8.8	−10.2	Referent	
Orsatti et al. [25], 2008					
Exercise	21.5	23.2		*P* = 0.56	
Control	25.1	27.4		Referent	
PATH trial [22, 23], 2004					
Exercise	18.3	17.5	−4.4	*P* = 0.32	
Control	17.9	17.8	−0.6	Referent	
Estrone (pg/ml)					
SHAPE-2 [18], 2015					
Exercise + diet	19.9	18.5	−6.67	0.92 (0.82:1.02)	Ex+WL vs WL
Reduced calorie diet	20.4	20.1	−1.26	0.98 (0.88:1.08)	0.94 (0.86:1.02)
Control	20.1	20.4	3.11	Referent	
NEW trial [17], 2012					
Exercise + diet	33.9	30.2	−11.1	*P* < 0.001	Ex+WL vs WL, *P* = 0.17
Exercise	34.8	32.9	−5.5	*P* < 0.01	Ex+WL vs Ex, *P* = 0.1
Reduced calorie diet	35.2	31.8	−9.6	*P* < 0.001	Ex vs WL, *P* = 0.3
Control	32.0	34.6	8.1	Referent	
ALPHA trial [24], 2010	31.4	29.4			
Exercise	31.3	30.6		0.99 (0.94–1.03)	
Control				Referent	
SHAPE-1 [3], 2009					
Exercise	30.6	27.6	−9.7	0.97 (0.92–1.04)	
Control	28.0	27.3	−3.4	Referent	
PATH trial [22, 23], 2004					
Exercise	44.2	42.5	−1.8	*P* = 0.13	
Control	43.9	45.4	3.9	Referent	
Free estradiol (pg/ml)					
SHAPE-2 [18], 2015					
Exercise + diet	0.09	0.07	−19.1	0.77 (0.67–0.88)	Ex+WL vs WL
Reduced calorie diet	0.10	0.08	−17.7	0.80 (0.70–0.92)	0.96 (0.85–1.02)
Control	0.09	0.10	3.23	Referent	
NEW trial [17], 2012					
Exercise + diet	0.32	0.23	−26	*P* < 0.001	Ex+WL vs WL, *P* = 0.06
Exercise	0.30	0.29	−4.7	*P* = 0.08	Ex+WL vs Ex, *P* < 0.001
Reduced calorie diet	0.31	0.24	−21.4	*P* < 0.001	Ex vs WL, *P* < 0 .001
Control	0.30	0.33	6.3	Referent	
ALPHA trial [24], 2010					
Exercise	0.24	0.21		0.91 (0.87–0.96)	
Control	0.25	0.24		Referent	
SHAPE-1 [3], 2009					
Exercise	0.22	0.21	−7.3	1.00 (0.96–1.04)	
Control	0.25	0.23	−10.2	Referent	
PATH trial [22, 23], 2004					
Exercise	0.49	0.46	−6.2	*P* = 0.2	
Control	0.47	0.47	0.0	Referent	
Testosterone (pg/ml)					
SHAPE-2 [18], 2015					
Exercise + diet	186	172	−7.63	0.96 (0.87–1.05)	Ex+WL vs WL
Reduced calorie diet	197	189	−3.76	1.01 (0.92–1.10)	0.95 (0.88–1.02))
Control	194	186	4.07	Referent	
NEW trial [17], 2012					
Exercise + diet	239	225	−5.9	*P* = 0.02	Ex+WL vs WL, *P* = 0.07
Exercise	248	236	−4.9	*P* = 0.24	Ex+WL vs Ex, *P* = 0.24
Reduced calorie diet	239	236	−0.9	*P* = 0.4	Ex vs WL, *P* = 0.67
Control	228	232	1.8	Referent	
ALPHA trial [24], 2010					
Exercise	239	234		0.99 (0.95–1.03)	
Control	231	237		Referent	
SHAPE-1 [3], 2009					
Exercise	528	507	−4.0	0.98 (0.94–1.01)	
Control	535	526	−1.6	Referent	
PATH trial [22, 23], 2004					
Exercise	211	208		*P* = 0.94	
Control	223	218		Referent	
Androstenedione (pg/ml)					
SHAPE-2 [18], 2015					
Exercise + diet	573	488	−14.7	0.87 (0.76–1.00)	Ex+WL vs WL
Reduced calorie diet	562	537	−4.5	0.97 (0.85–1.12)	0.90 (0.80–1.01)
Control	575	560	−2.6	Referent	
NEW trial [17], 2012					
Exercise + diet	526	508	−3.5	*P* = 0.22	Ex+WL vs WL, *P* = 0.26
Exercise	502	496	−1.2	*P* = 0.75	Ex+WL vs Ex, *P* = 0.25
Reduced calorie diet	511	518	1.4	*P* = 0.83	Ex vs WL, *P* = 0.93
Control	487	494	1.5	Referent	
ALPHA trial [24], 2010					
Exercise	578	572		0.98 (0.93–1.03)	
Control	553	577		Referent	
SHAPE-1 [3], 2009					
Exercise	1146	1115	−2.7	0.97 (0.93–1.01)	
Control	1172	1199	2.3	Referent	
PATH trial [22, 23], 2004					
Exercise	533	480		*P* = 0.89	
Control	585	525		Referent	
Free testosterone (pg/ml)					
SHAPE-2 [18], 2015					
Exercise + diet	2.44	2.01	−17.7	0.84 (0.76–0.93)	Ex+WL vs WL
Reduced calorie diet	2.53	2.25	−11.2	0.91 (0.83–1.01)	0.92 (0.85–0.99)
Control	2.71	2.61	−3.9	Referent	
NEW trial [17], 2012					
Exercise + diet	5.3	4.5	−15.6	*P* < 0.001	Ex+WL vs WL, *P* = 0.02
Exercise	5.1	4.9	−4.5	*P* = 0.2	Ex+WL vs Ex, *P* < 0.001
Reduced calorie diet	5.1	4.6	−10.0	*P* < 0.001	Ex vs WL, *P* = 0.02
Control	4.9	5.1	2.6	Referent	
ALPHA trial [24], 2010					
Exercise	3.5	3.3		0.96 (0.92–1.01)	
Control	3.5	3.5		Referent	
SHAPE-1 [3], 2009					
Exercise	8.7	8.5	−2.9	0.99 (0.95–1.03)	
Control	8.7	8.5	−1.8	Referent	
PATH trial [22, 23], 2004					
Exercise	4.6	4.3		*P* = 0.42	
Control	4.7	4.6		Referent	
SHBG (nmol/l)					
SHAPE-2 [18], 2015					
Exercise + diet	49.3	58.6	19.0	1.21 (1.12–1.30)	Ex+WL vs WL
Reduced calorie diet	50.7	57.1	12.6	1.14 (1.07–1.23)	1.05 (1.00–1.12)
Control	44.2	44.0	−0.30	Referent	
NEW trial [17], 2012					
Exercise + diet	34.1	42.9	25.8	*P* < 0.001	Ex+WL vs WL, *P* = 0.41
Exercise	39.1	38.8	0.7	*P* = 0.41	Ex+WL, vs Ex *P* < 0.001
Reduced calorie diet	35.8	43.8	22.4	*P* < 0.001	Ex vs WL, *P* < 0.001
Control	34.7	33.7	−2.7	Referent	
ALPHA trial [24], 2010					
Exercise	40.3	41.9		1.04 (1.02–1.07)	
Control	38.1	38.4		Referent	
SHAPE-1 [3], 2009					
Exercise	33.9	33.6	−0.7	0.98 (0.92–1.04)	
Control	34.7	33.6	−3.3	Referent	
PATH trial [22, 23], 2004					
Exercise	35.2	38.3	8.8	*P* = 0.10	
Control	35.8	36.7	2.5	Referent	

*ALPHA* Alberta Physical Activity and Breast Cancer, *NEW* Nutrition and Exercise for Woman, *PATH* Physical Activity for Total Health, *SHAPE* Sex Hormone and Physical Exercise, *SHBG* sex hormone binding globulin

^a^Geometric means reported

^b^Values are given as either treatment effect ratios (95% confidence intervals) or as *P* values

^c^Body mass index (BMI) was reported when bodyweight was not available

### Sex hormone levels

Five studies reported geometric means for the relevant sex hormone levels (i.e., total estradiol, free estradiol, estrone, SHBG, total testosterone, free testosterone, and androstenedione) [3, 17, 18, 22–24]. One study reported data only on total estradiol and total testosterone (means not based on log transformed data) and no other sex hormones [25]. The reported measure of association varied by trial. Both the SHAPE trials [3, 18] and the ALPHA trial [24] reported absolute change, percentage change, TER, and the 95% CI of the TER. Both the PATH and NEW trials reported absolute change, percentage change, and the *p* values for between-group differences [17, 22, 23]. The study of Orsatti et al. could technically not be included in the meta-analysis because arithmetic means were reported and geometric means could not be re-estimated [25].

**Fig. 2:**
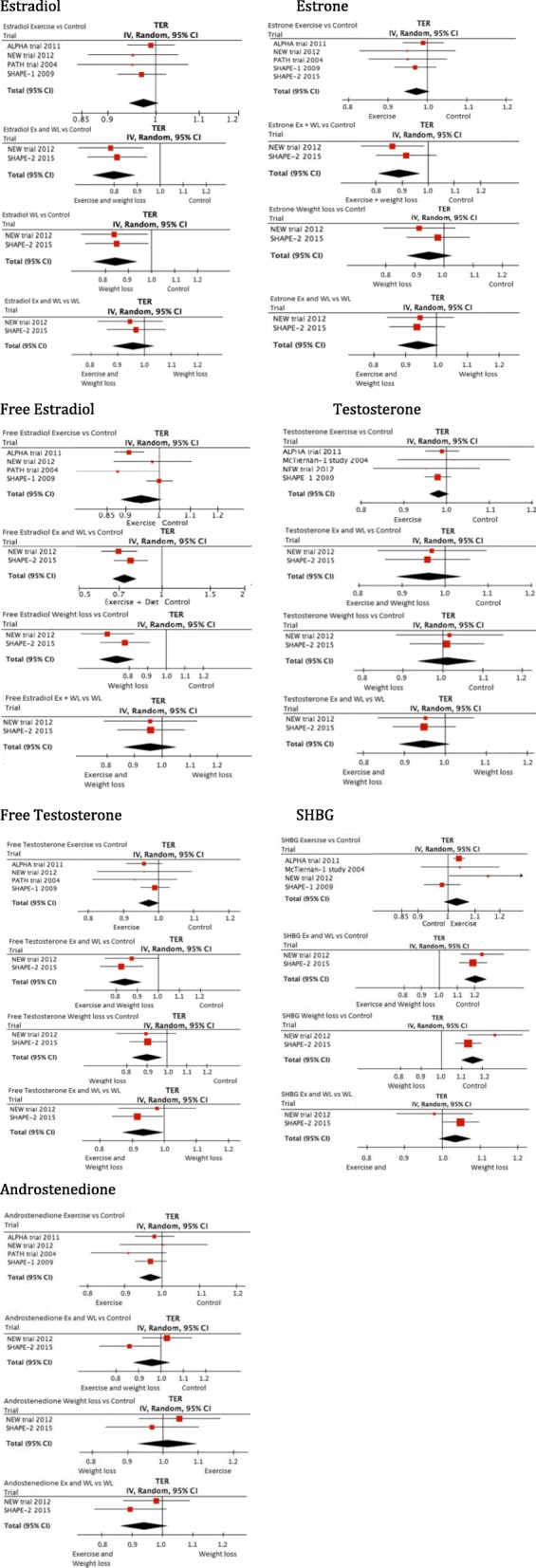
Forest plots per sex hormone. Plots per comparison: 1) exercise compared with control; 2) exercise (Ex) and diet (WL) versus control; 3) diet (WL) versus control; 4) exercise (Ex) and diet (WL) versus diet (WL). ALPHA Alberta Physical Activity and Breast Cancer, CI confidence interval, NEW Nutrition and Exercise for Woman, PATH Physical Activity for Total Health, SHAPE Sex Hormone and Physical Exercise, TER treatment effect ratio

Table 4, Fig. 2, and Additional file 3 show the treatment effects and CIs of all our analyses. Below, we describe our results. We report only statistically significant TERs and 95% CIs.

**Table 4:** Pooled mean differences of the four comparisons on the different sex hormone outcomes and sex hormone binding globulin (SHBG)

Pooled effects^a^	Treatment effect ratios (95% confidence interval)
Estrone	
Exercise vs control	0.97 (0.94–1.01)
Exercise + diet vs control	0.90 (0.83–0.97)
Diet vs control	0.95 (0.88–1.03)
Exercise + diet vs diet	0.94 (0.88–1.01)
Total estradiol	
Exercise vs control	0.97 (0.94–1.00)
Exercise + diet vs control	0.82 (0.75–0.90)
Diet vs control	0.86 (0.77–0.95)
Exercise + diet vs diet	0.96 (0.89–1.04)
Free estradiol	
Exercise vs control	0.95 (0.87–1.01)
Exercise + diet vs control	0.73 (0.66–0.81)
Diet vs control	0.77 (0.69–0.84)
Exercise + diet vs diet	0.96 (0.87–1.06)
Total testosterone	
Exercise vs control	0.98 (0.95–1.01)
Exercise + diet vs control	0.96 (0.89–1.04)
Diet vs control	1.01 (0.94–1.09)
Exercise + diet vs diet	0.95 (0.89–1.01)
Free testosterone	
Exercise vs control	0.97 (0.95–1.00)
Exercise + diet vs control	0.86 (0.79–0.93)
Diet vs control	0.91 (0.84–0.98)
Exercise + diet vs diet	0.94 (0.88–1.00)
Androstenedione	
Exercise vs control	0.97 (0.94–1.00)
Exercise + diet vs control	0.95 (0.80–1.12)
Diet vs control	1.01 (0.93–1.11)
Exercise + diet vs diet	0.94 (0.87–1.02)
SHBG	
Exercise vs control	1.03 (0.99–1.08)
Exercise + diet vs control	1.23 (1.15–1.31)
Diet vs control	1.20 (1.06–1.36)
Exercise + diet vs diet	1.03 (0.97–1.09)

^a^ For readability reduced calorie diet is labeled as “diet”

### Exercise versus control

Four studies compared an exercise intervention with no intervention (Table 3) [3, 17, 22–24]. Pooled TERs were borderline statistically significant for androstenedione (0.97, 95% CI 0.94–1.00; *P* = 0.05), for total estradiol (0.97, 95% CI 0.94–1.00; *P* = 0.06), and free testosterone (0.97, 95% CI 0.95–1.00; *p* = 0.09) in favor of the exercise group (Table 4 and Fig. 2). Pooled TERs for estrone, free estradiol, total testosterone, and SHBG were in favor of the exercise group, although not statistically significant.

### Combined exercise and reduced calorie diet versus control

Two studies compared combined reduced calorie diet and exercise interventions versus controls [17, 18]. The control groups in both studies were requested not to change their diet (NEW trial) [17] or follow a standardized diet (SHAPE-2) and maintain their exercise habits [18]. Both control groups were offered alternative weight loss programs after study completion. Pooled TERs showed a statistically significant effect for total estradiol (0.82, 95% CI 0.75–0.90), for free estradiol (0.73, 95% CI 0.66–0.81), for estrone (0.90, 95% CI 0.83–0.97), for free testosterone (0.86, 95% CI 0.79–0.93), and for SHBG (1.23, 95% CI 1.15–1.31) in favor of the combined exercise and reduced calorie intervention. Pooled effects for total testosterone showed a favorable effect for the exercise and reduced calorie group, although this was not statistically significant. No statistically significant effects were found for androstenedione.

### Reduced calorie diet versus control

Meta-analysis of two studies resulted in a statistically significant decrease in favor of the reduced calorie group for total estradiol (0.86, 95% CI 0.77–0.95), for free estradiol (0.77, 95% CI 0.69–0.84), for free testosterone (0.91, 95% CI 0.84–0.98), and an increase for SHBG (1.20, 95% CI 1.06–1.36), and a favorable but not statistically significant decrease in estrone [17, 18]. No statistically significant effects were found for total testosterone and androstenedione.

### Combined exercise and reduced calorie diet versus diet

Meta-analysis of two studies showed a statistically significant decrease in free testosterone (0.94, 95% CI 0.88–1.00) for a combination of exercise and reduced calorie diet compared with reduced calorie diet only. A favorable decrease, although not statistically significant, was shown for estrone (0.94, 95% CI 0.88–1.01), total testosterone (0.95, 95% CI 0.89–1.01), and androstenedione (0.94, 95% CI 0.87–1.02) [17, 18]. No statistically significant effects were found on SHBG, total, or free estradiol.

### Combined exercise and reduced calorie diet versus exercise

One study compared exercise combined with a reduced calorie diet to exercise alone [17]. Since only one study performed this comparison, original study data are shown instead of estimating the TER. When compared with the exercise-only intervention, the exercise combined with a reduced calorie intervention showed significant beneficial changes for estrone (−1.9 pg/ml, *P* = 0.01), total estradiol (−1.7 pg/ml, *P* < 0.001), free estradiol (−0.07 pg/ml, *P* < 0.01), SHBG (+9.1 nmol/l, *P* = < 0.01), and free testosterone (−0.59 pg/ml, *P* < 0.01) [17]. For total testosterone and androstenedione no statistically significant results were found [17].

### Exercise versus reduced calorie diet

This comparison was also only investigated in one study [17]. The reduced calorie intervention showed beneficial statistically significant results when compared with the exercise intervention for total estradiol (−1.3 pg/ml, *P* = 0.002), free estradiol (−0.06 pg/ml, *P* < 0.001), free testosterone (−0.28 pg/ml, *P* = 0.02), and SHBG (+8.3 nmol/l, *P* < 0.001) [17]. No statistically significant effects were found for estrone, total testosterone, or androstenedione [17].

## Discussion

This systematic review and meta-analysis found beneficial effects on endogenous estrogen levels and free testosterone from interventions that were designed to change either dietary caloric intake, exercise levels, or both, in postmenopausal healthy women, which is relevant for breast cancer risk reduction in this population. No beneficial effects were found for any of these interventions on total testosterone levels (only in free testosterone). Our meta-analysis suggests that weight loss is important for achieving effects on hormone levels, and caloric restriction (with or without an exercise component) affects weight loss to a larger extent than exercise only in physically inactive postmenopausal women. We found that caloric restriction combined with exercise seems to be most beneficial for lowering sex hormone levels. Comparing the combination of exercise and caloric restriction with caloric restriction only, all results favored the combination even when weight loss between the groups was comparable. An additional important advantage of combining caloric restriction with exercise is that the exercise component maintains or increases muscle mass and cardiovascular fitness.

The studies in this meta-analysis mostly showed beneficial effects of exercise and/or caloric restriction on endogenous sex hormones, although the magnitude of effects varied. There are several underlying factors that can explain this variation. First, varying types, doses, and duration of interventions might be responsible for differences between studies. Second, inclusion criteria across studies were largely comparable, but differences in baseline BMI and other differences in study populations might have contributed to varying results on endogenous sex hormones. The SHAPE-1, the ALPHA trial, and the study of Orsatti et al. included normal-weight women [3, 24, 25], while the other studies excluded these women. Women with normal weight might have less room for improvement in sex hormone levels since this change depends on the amount of fat mass. Similarly, although all studies included “inactive” women, the definition of “inactive” varied between studies. Third, the studies varied by the mean weight loss in the intervention group(s), with larger weight loss in the studies that explicitly aimed for weight loss. On average, stronger effects were found in the NEW trial and in the SHAPE-2 study [17, 18]. Contrary to the ALPHA, PATH, and SHAPE-1 trials, the interventions in the NEW and SHAPE-2 trials targeted weight loss, with goals of −10% of body weight and 5 to 6 kg, respectively [3, 17, 18, 22–24]. This difference might explain the larger effects since all studies found that women who lost larger amounts of weight showed larger effects on sex hormone levels [16, 28, 29]. Results of the trials studying the effect of exercise without aiming for weight loss show that exercise only is not sufficient to affect the hormone levels substantially [3, 17, 18, 22–24]. After stratifying for fat loss, the SHAPE-1 and PATH trials both reported larger effects on hormone levels in women who lost > 2% of body fat [3, 22, 23]. Hence, it is important to achieve weight loss to affect sex hormone levels [16, 28, 29]. Pooled effects for diet (compared with control groups) showed statistically significant results for several hormones, which was not observed for interventions with mainly exercise.

Although our meta-analysis showed beneficial effects of exercise and/or caloric restriction on most endogenous sex hormones, null associations were found for total testosterone. This result was an unexpected finding because of the earlier observed associations between increased adiposity and increased androgen levels and because of effects for free testosterone that were statistically significant [30–32]. A potential reason for this null association might be the large variation in testosterone values and the extremely low levels, which complicate detecting effects.

It is still a challenge to estimate the magnitude of the clinical impact of the observed effects on sex hormones, since there are no absolute cut-off values defined that correspond with a certain change in future breast cancer risk. Until now, it is assumed that the distributions and rankings of sex hormone levels, rather than the absolute values, correspond with breast cancer risk. Observational studies that linked sex hormone levels to breast cancer risk mainly show that women whose hormone levels are in the highest quintiles of the distribution have an up to twofold increased risk when compared with women with levels in the lowest quintiles [12, 33]. However, the absolute values corresponding to these quintiles vary largely between studies. For example, the Endogenous Hormones and Breast Cancer Collaborative Group evaluated nine prospective studies that measured sex hormones in postmenopausal breast cancer cases and samples of healthy postmenopausal controls [12]. Median hormone levels varied substantially; for example, estradiol levels differed up to fivefold between the studies, ranging from 22 pmol/l to 101 pmol/l in control women. Besides population heterogeneity (in ages, BMI, and other determinants of hormone levels such as reproductive factors and nutritional habits), the large variation in absolute values is probably mainly caused by differences in laboratory assays [34, 35]. These issues might, in addition to the different intervention programs, explain the differences in magnitude of effects across the studies included in this meta-analysis.

The focus of this meta-analysis is on breast cancer-related endogenous sex hormones, but there might be additional beneficial effects of adding exercise to a dietary intervention. It has been shown that exercise interventions have beneficial effects on cardiopulmonary fitness, may prevent diabetes, increase muscular strength, and lower the risk of osteoporosis. For example, the SHAPE-2 study showed a small loss of muscle mass in the reduced calorie group, which should be avoided [18]. Therefore, including an exercise component in the intervention is highly recommended rather than a reduction in caloric intake alone.

The strength of this meta-analysis is that the separate trials were each of high quality with large sample sizes. This meta-analysis also has some limitations. First, results might not be generalizable to all postmenopausal women, since only physically inactive women with a BMI > 22 kg/m^2^ were included in this meta-analysis. We were not able to stratify our results in this meta-analysis for physical activity levels because the interventions differed in duration, intensity, and type of exercises.

There are several topics for further research. First, studies considering the long-term maintenance of the effect on endogenous sex hormones are lacking. For the sustainability of intervention effects, behavioral changes in food intake and daily physical activity are necessary. A follow-up study from the SHAPE-2 trial found that the participants were able to maintain weight loss and increase physical activity levels in both study groups 1 year after trial completion, but sex hormone levels were not measured again at the 1-year follow-up time point [36]. A second topic of interest is whether or not the effects are found in different population subgroups, such as women of different race/ethnic origin, or women at risk for breast cancer because of familial predisposition (e.g., breast cancer (BRCA)1 and BRCA2 genes). Third, future research should consider different biologic mechanisms that have not yet been investigated, such as immune function.

## Conclusions

In conclusion, the combined data from six randomized controlled trials demonstrate that there are beneficial effects when weight loss was achieved by a reduced calorie diet intervention with or without exercise on breast cancer-related endogenous sex hormones in overweight, physically inactive postmenopausal women. Our results suggest that the most beneficial effects on endogenous sex hormones were found with a combined exercise and reduced caloric dietary intervention. Exercise interventions without a reduced caloric intake showed small effects on endogenous sex hormone levels. To reduce breast cancer-related endogenous sex hormones, we recommend combining a reduced calorie diet with exercise to increase weight loss and maintain or increase muscle mass and cardiovascular fitness.

## Additional files

Additional file 1: Search string in PubMed. The search string we used in PubMed in this meta-analysis. (DOCX 103 kb)

Additional file 2: Cochrane bias tool. The Cochrane’s collaboration risk of bias tool we used for assessing the risk of bias for the included studies. (DOCX 145 kb)

Additional file 3: Forest plots with treatment effect ratios (TERs). The forest plots with the associated treatment effect ratios per intervention group per study. (DOCX 1392 kb)

## Abbreviations

ALPHA: Alberta Physical Activity and Breast Cancer
BMI: Body mass index
CI: Confidence interval
HRR: Heart rate reserve
MHR: Maximum heart rate
NEW: Nutrition and Exercise for Woman
PATH: Physical Activity for Total Health
RCT: Randomized controlled trial
SE: Standard error
SHAPE: Sex Hormone and Physical Exercise
SHBG: Sex hormone binding globulin
TER: Treatment effect ratio
