# Supplementary material for: Effect of exercise and/or reduced calorie dietary interventions on breast cancer-related endogenous sex hormones in healthy postmenopausal women
Source: Breast Cancer Res. 2018 Aug 2;20:81. doi: 10.1186/s13058-018-1009-8 (PMC6090977; doi:10.1186/s13058-018-1009-8)
Supplement: Supplementary file 1 — Search string in PubMed. The search string we used in PubMed in this meta-analysis. (DOCX 103 kb) [file 13058_2018_1009_MOESM1_ESM.docx]

**Additional file 1**: Search string PubMed

(((((((((((((((((((((((((((((((motor activity[MeSH Terms]) OR physical activity[Title/Abstract]) OR physical activities[Title/Abstract]) OR activities, physical[MeSH Terms]) OR exercise[MeSH Terms]) OR exercise[Title/Abstract]) OR aerobic exercise[MeSH Terms]) OR aerobic exercise[Title/Abstract]) OR aerobic training[Title/Abstract])OR strength training[MeSH Terms]) OR strength training[Title/Abstract]) OR endurance training[Title/Abstract]) OR endurance exercise[Title/Abstract]) OR fitness exercise[Title/Abstract]) OR fitness training[Title/Abstract]) OR exercise intervention[Title/Abstract]) OR strength exercises[Title/Abstract]) OR physical training[Title/Abstract]) OR motor activity[title/Abstract]))

OR

(((((((((((((((((weight loss[MeSH Terms]) OR diet, weight loss[MeSH Terms]) OR program, weight loss[MeSH Terms]) OR weight loss[Title/Abstract]) OR body weight change[MeSH Terms]) OR diet, weight reduction[MeSH Terms])OR program, weight reduction[MeSH Terms]) OR weight reduction diet[MeSH Terms]) OR body weight change[Title/Abstract]) OR body weight changes[Title/Abstract]) OR weight reduction[Title/Abstract]) OR weight reductions[Title/Abstract]) OR caloric restriction[MeSH Terms]) OR caloric restriction[Title/Abstract]) OR hypocaloric diet[Title/Abstract])))))

AND

(((((postmenopause[MeSH Terms]) OR post menopausal[Title/Abstract]) OR Post-menopausal[Title/Abstract]) OR Postmenopausal[Title/Abstract]) OR Postmenopause[Title/Abstract]))

AND

((((((((((((((((((((((((((sex hormones[MeSH Terms]) OR sex hormones[Title/Abstract]) OR sex hormone binding globulin[MeSH Terms]) OR sex hormone binding globulin[Title/Abstract]) OR globulin, sex hormone binding[MeSH Terms]) OR adipokine[MeSH Terms]) OR adipokine[Title/Abstract]) OR cytokines[MeSH Terms]) OR cytokines[Title/Abstract]) OR estrogens[MeSH Terms]) OR estrogens[Title/Abstract]) OR oestradiol[MeSH Terms]) OR oestradiol[Title/Abstract]) OR androgens[MeSH Terms]) OR androgens[Title/Abstract]) OR leptin[MeSH Terms]) OR leptin[Title/Abstract]) OR testosterone[MeSH Terms]) OR testosterone[Title/Abstract]) OR insulin[MeSH Terms]) OR insulin[Title/Abstract]) OR biomarkers[Title/Abstract]) OR estradiol[Title/Abstract]) OR inflammatory markers[Title/Abstract]) OR shbg[Title/Abstract] OR biological markers[MeSH Terms])))
