# Supplementary material for: Effect of exercise and/or reduced calorie dietary interventions on breast cancer-related endogenous sex hormones in healthy postmenopausal women
Source: Breast Cancer Res. 2018 Aug 2;20:81. doi: 10.1186/s13058-018-1009-8 (PMC6090977; doi:10.1186/s13058-018-1009-8)

**Additional file 2**: Cochrane bias tool

Figure 2: Risk of bias summary: review authors' judgements about each risk of bias item for each included study.

*For other bias three topics were scored: Were blood samples of the same women analysed in the same batch, were participants instructed to avoid exercise 24 hours before blood sampling, and was adherence to the exercise programme monitored. If one of these three items was not performed we scored this topic as high risk of bias (red), if it was not described as unclear (yellow), and when all topics were described as low risk of bias (green).


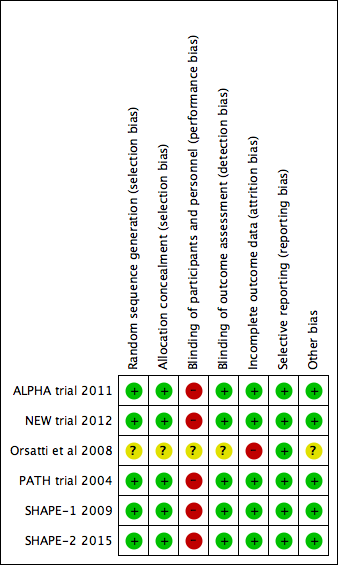

Supplement: Supplementary file 2 — Cochrane bias tool. The Cochrane’s collaboration risk of bias tool we used for assessing the risk of bias for the included studies. (DOCX 145 kb) [file 13058_2018_1009_MOESM2_ESM.docx]
