# Supplementary material for: Effect of exercise and/or reduced calorie dietary interventions on breast cancer-related endogenous sex hormones in healthy postmenopausal women
Source: Breast Cancer Res. 2018 Aug 2;20:81. doi: 10.1186/s13058-018-1009-8 (PMC6090977; doi:10.1186/s13058-018-1009-8)

**Additional file 3:** Forest plots with treatment effect ratios (TER)

Estrone


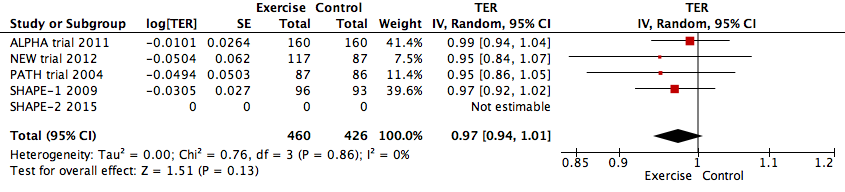


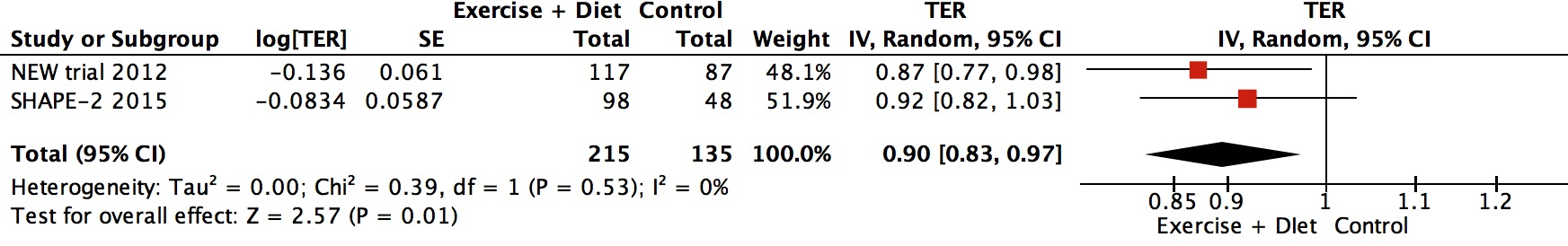


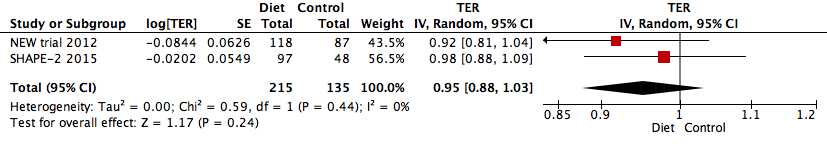


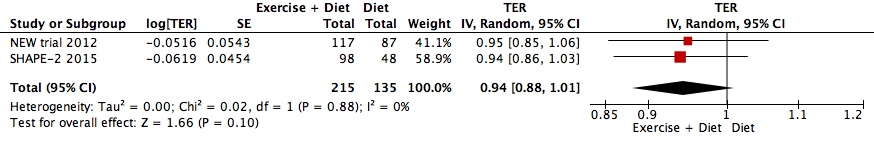


Estradiol


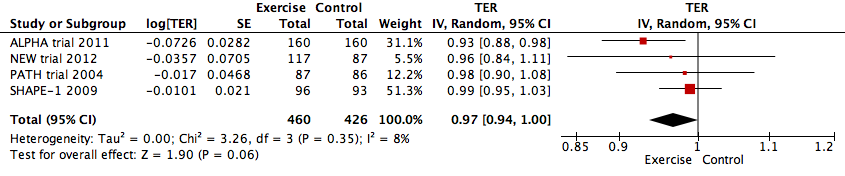


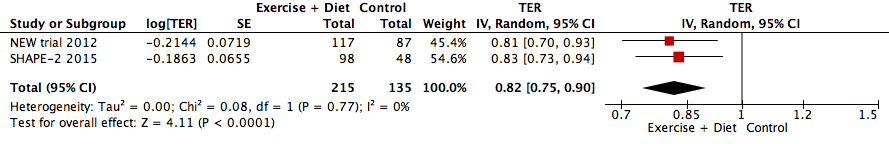


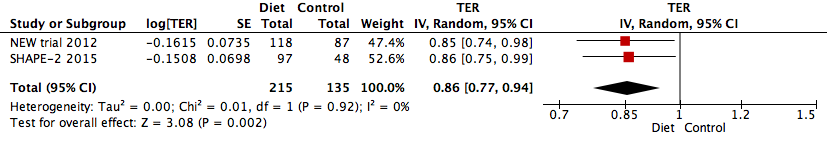


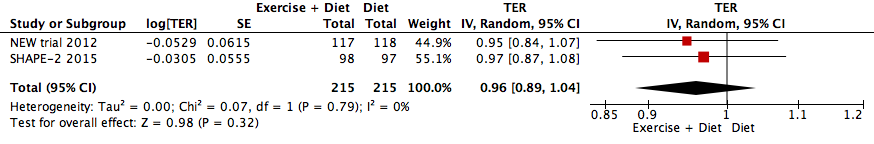


Free Estradiol


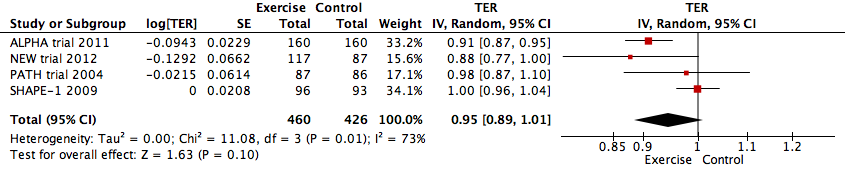


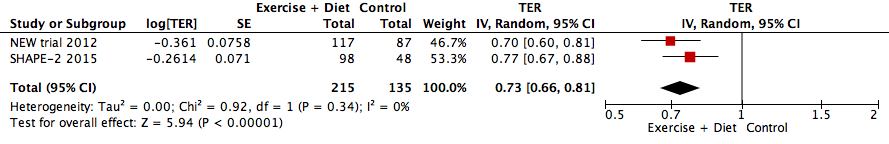


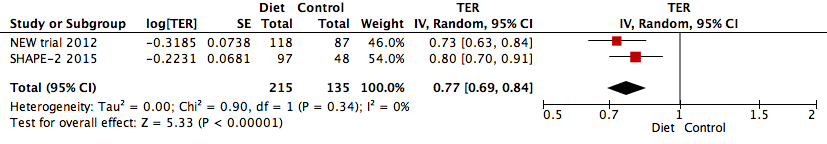


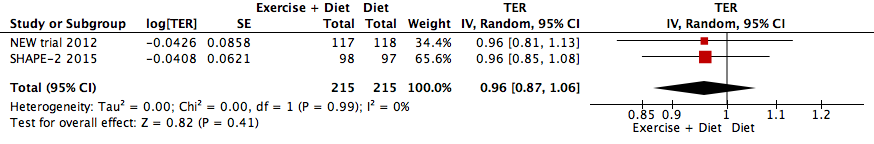


Testosterone


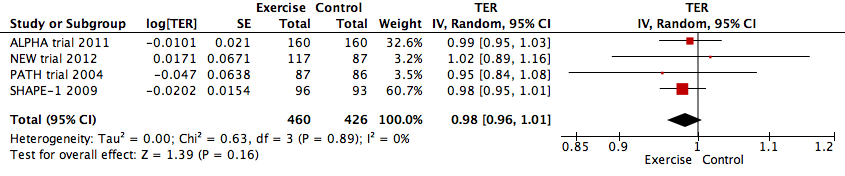


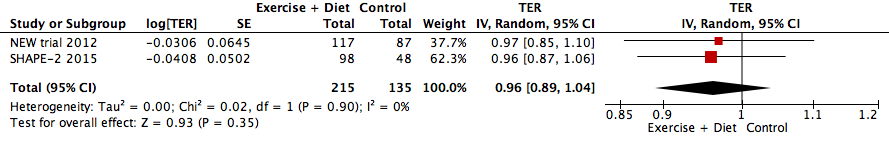


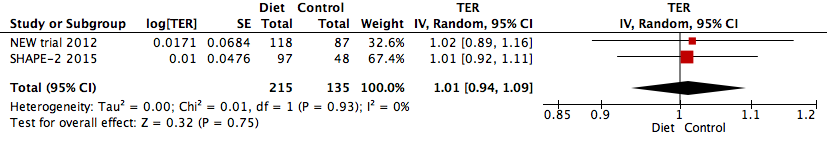


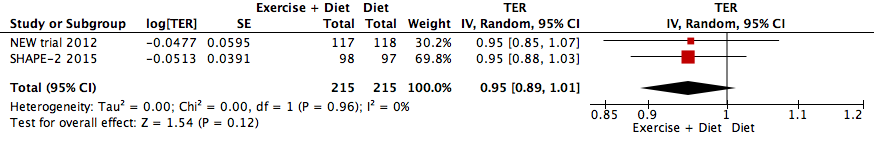


Free Testosterone


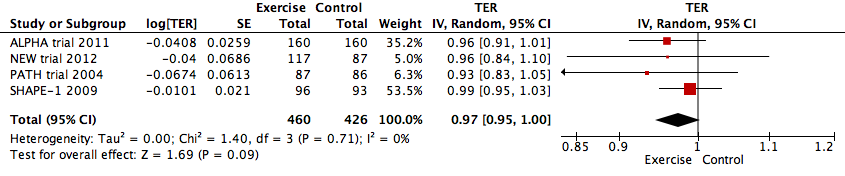


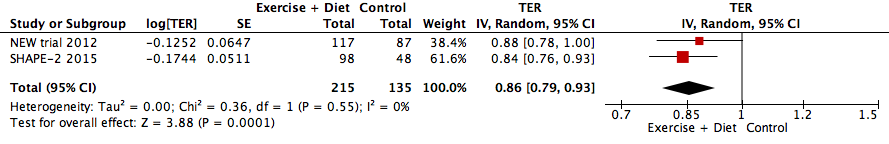


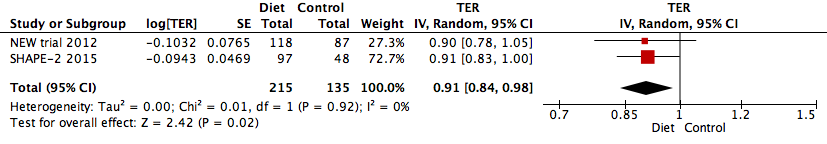


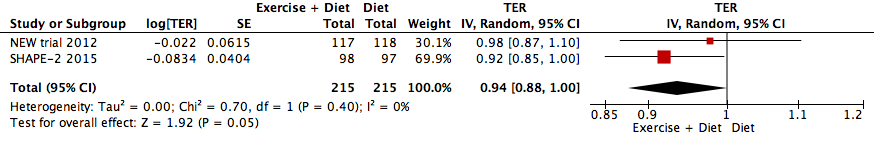


SHBG


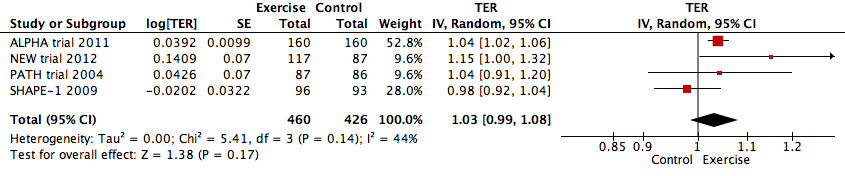


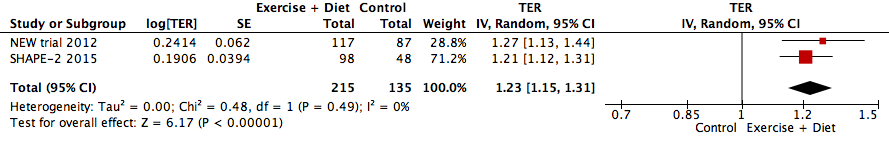


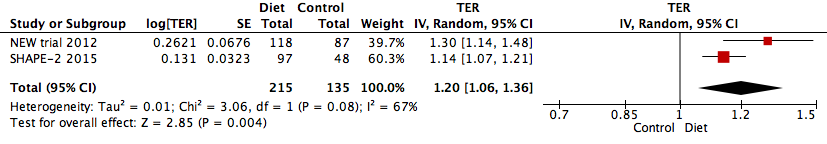


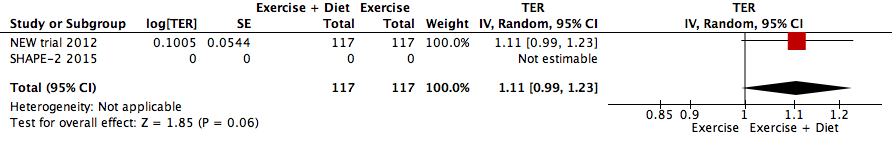


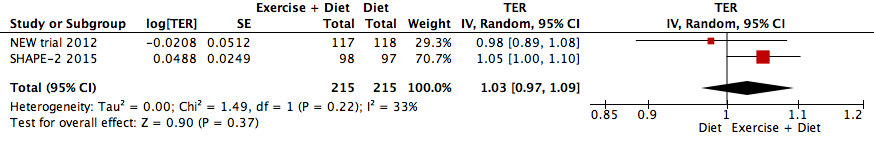


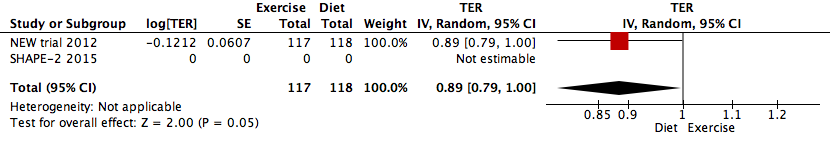


Androstenedione


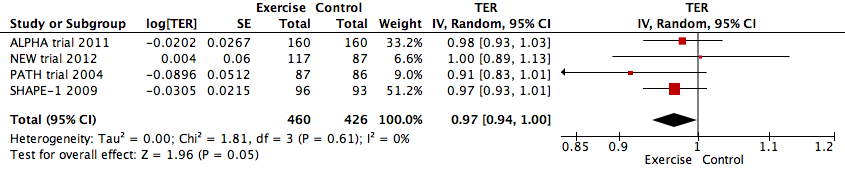


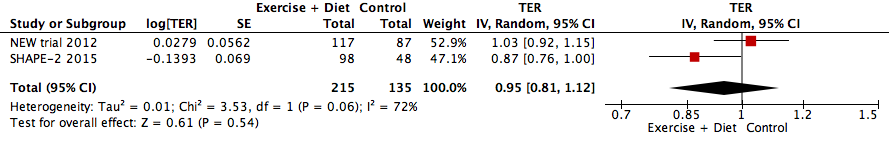


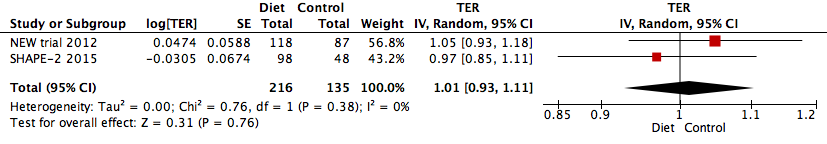


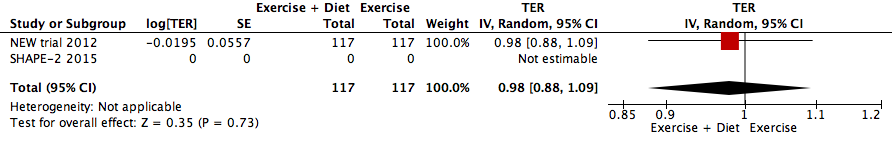


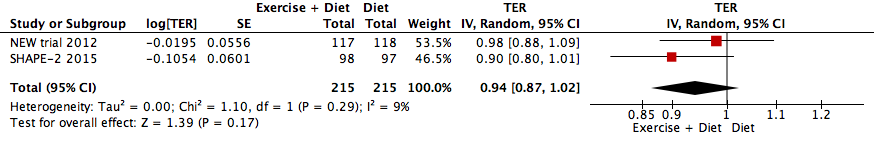


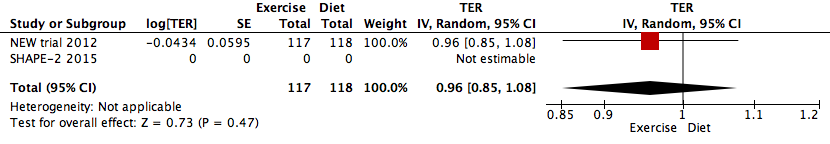

Supplement: Supplementary file 3 — Forest plots with treatment effect ratios (TERs). The forest plots with the associated treatment effect ratios per intervention group per study. (DOCX 1392 kb) [file 13058_2018_1009_MOESM3_ESM.docx]
